# Predicting urinary tract infections in the emergency department with machine learning

**DOI:** 10.1371/journal.pone.0194085

**Published:** 2018-03-07

**Authors:** R. Andrew Taylor, Christopher L. Moore, Kei-Hoi Cheung, Cynthia Brandt

**Affiliations:** Department of Emergency Medicine, Yale University School of Medicine, New Haven CT, United States of America; University of North Texas, UNITED STATES

## Abstract

**Background:**

Urinary tract infection (UTI) is a common emergency department (ED) diagnosis with reported high diagnostic error rates. Because a urine culture, part of the gold standard for diagnosis of UTI, is usually not available for 24–48 hours after an ED visit, diagnosis and treatment decisions are based on symptoms, physical findings, and other laboratory results, potentially leading to overutilization, antibiotic resistance, and delayed treatment. Previous research has demonstrated inadequate diagnostic performance for both individual laboratory tests and prediction tools.

**Objective:**

Our aim, was to train, validate, and compare machine-learning based predictive models for UTI in a large diverse set of ED patients.

**Methods:**

Single-center, multi-site, retrospective cohort analysis of 80,387 adult ED visits with urine culture results and UTI symptoms. We developed models for UTI prediction with six machine learning algorithms using demographic information, vitals, laboratory results, medications, past medical history, chief complaint, and structured historical and physical exam findings. Models were developed with both the full set of 211 variables and a reduced set of 10 variables. UTI predictions were compared between models and to proxies of provider judgment (documentation of UTI diagnosis and antibiotic administration).

**Results:**

The machine learning models had an area under the curve ranging from 0.826–0.904, with extreme gradient boosting (XGBoost) the top performing algorithm for both full and reduced models. The XGBoost full and reduced models demonstrated greatly improved specificity when compared to the provider judgment proxy of UTI diagnosis OR antibiotic administration with specificity differences of 33.3 (31.3–34.3) and 29.6 (28.5–30.6), while also demonstrating superior sensitivity when compared to documentation of UTI diagnosis with sensitivity differences of 38.7 (38.1–39.4) and 33.2 (32.5–33.9). In the admission and discharge cohorts using the full XGboost model, approximately 1 in 4 patients (4109/15855) would be re-categorized from a false positive to a true negative and approximately 1 in 11 patients (1372/15855) would be re-categorized from a false negative to a true positive.

**Conclusion:**

The best performing machine learning algorithm, XGBoost, accurately diagnosed positive urine culture results, and outperformed previously developed models in the literature and several proxies for provider judgment. Future prospective validation is warranted.

## Introduction

In the United States, there are more than 3 million emergency department (ED) visits each year for urinary tract infections (UTI) with annual direct and indirect costs estimated to be more than $2 billion.[1–3] Compared with the general population, ED patients with UTIs have higher acuity (approximately 10% of visits are for pyelonephritis) and are more likely to present with non-classic symptoms such as altered mental status, fatigue, and nausea.[4] Because a urine culture, part of the gold standard for diagnosis of UTI, is usually not available for 24–48 hours after an ED visit, diagnosis and treatment decisions are based on symptoms, physical findings, and other laboratory results, potentially leading to overutilization, antibiotic resistance, and delayed treatment. [5]

Diagnostic error for UTI in the ED has been reported to be as high as 30–50%.[6–8] While women of child-bearing age exhibiting classic symptoms of dysuria, frequency, and hematuria have a high likelihood of disease, in more generalized cohorts of ED patients historical, physical, and laboratory findings are less accurate.[9, 10] In a systematic review of ED studies pertaining to urinalysis results, Meister et al. found that only the presence of nitrite was specific enough to rule in the disease, while no single test or simple combination of tests was able to rule out the disease.[10] Furthermore, many of these prior studies examining UTI focused on high prevalence populations with uncomplicated UTI, creating concern for spectrum bias in the results.[11] These findings have led to calls for development of more sophisticated clinical decision support systems with predictive models that incorporate multiple aspects of both history, physical, and laboratory findings to improve diagnostic accuracy.[10]

While some predictive models for UTI have been developed, [12–17] they are limited in several ways. Most use only a few variables (e.g. only urine dipstick or urinalysis results), were derived from small datasets, and fail to model for complex interactions between variables which results in poor to moderate diagnostic performance. Others, like the neural network developed by Heckerling et al.[16], have improved diagnostic accuracy but were derived on female-only data sets of generally healthy outpatient populations with high prevalences of UTI, limiting their generalizability. Yet, now with the recent widespread adoption of Electronic Health Records (EHRs) and advances in data science[18], there is the opportunity to move beyond these limited predictive models and develop and deploy sophisticated machine learning algorithms, trained on thousands to millions of examples to assist with UTI diagnosis and potentially reduce diagnostic error.

Our aim, therefore, was to train, validate, and compare predictive models for UTI in a diverse set of ED patients using machine learning algorithms on a large single-center, multi-site, electronic health record (EHR) dataset. Within the validation dataset, we further sought to compare the best performing model to proxies of clinical judgement by examining provider patterns of UTI diagnosis and antibiotic prescription to gain insight about the potential impact of the model.

## Methods

### Design

Single-center, multi-site, retrospective cohort analysis of adult emergency department visits with urine culture results. This study was approved by the institutional review board (Yale Human Research Protection Program) and waived the requirement for informed consent. Data were de-identified after initial database access, but prior to analysis. Only de-identified data was stored and used in analyses (see S1 File for minimal data set and S2 File for code used in analyses). We adhered to the Transparent Reporting of a multivariable prediction model for Individual Prognosis or Diagnosis (TRIPOD) statement on reporting predictive models.[19]

### Study setting and population

Data were obtained from four EDs between March 2013 and May 2016. All EDs were part of a single health care system and have been described previously.[20] All EDs use a single EHR vendor, Epic (Verona, WI) with a centralized data warehouse. We included all visits for adult patients (≥18 years) who had a urine culture obtained during their ED visit and who had symptoms potentially attributable to a UTI (Table 1). The requirement to have symptoms potentially attributable to UTI was made to eliminate visits where patients may have asymptomatic bacteriuria.[21]

**Table 1:** Signs and symptoms potentially attributable to UTI*.

Chief Complaints
Abdominal Pain
Genitourinary Problem
Urinary Tract Infection
Altered Mental Status
Fever
Hematuria
Flank Pain
Dysuria
Symptoms
Altered Mental Status
Pelvic Pain
Difficulty Urinating
Flank Pain
Abdominal Pain
Dysuria
Polyuria
Hematuria
Fever
Signs
Costovertebral Angle Tenderness
Abdominal Tenderness
Abdominal Guarding
Abdominal Rigidity

* Incorporated as part of inclusion criteria to exclude patients with asymptomatic bacteriuria

### Data set creation and definitions

All data elements for each ED visit were obtained from the enterprise data warehouse. Only data available during the ED visit until the time of admission or discharge were used as prediction variables. Medications received during the ED visit and ED diagnosis were not included as variables to eliminate the influence of provider knowledge on the prediction model. Predictor variables included demographic information (age, sex, race, etc.), vitals, laboratory results, urinalysis and urine dipstick results, current outpatient medications, past medical history, chief complaint, and structured historical and physical exam findings (S1 Table).

### Data preprocessing

Data were preprocessed according to methods previously described.[20] Errant text data in categorical fields were improved through regular expression searches. Continuous data (labs, vitals) within the EHR are often not missing at random and provide additional information if encoded in some way. For example, in patients who are viewed as “not sick” labs are often not ordered. Continuous data were therefore smoothed and discretized using k-means clustering (k value = 5) allowing incorporation of a “not recorded” category.[22] Medications and comorbidities were grouped using the Anatomical Therapeutic Chemical (ATC) Classification System and Clinical Classification Software categories[23, 24]

### Outcomes

The primary outcome for all analyses was the presence of a positive urine culture defined by >10^4^ colony forming units (CFU)/high powered field (HPF), a threshold pre-established by the laboratory of our healthcare system for reporting positive results. Mixed flora results were only considered positive if there was the presence of Escherichia coli.[25] For the secondary aim, we compared the best performing model to clinical judgement. While EHR data readily allows the accumulation of large amounts of data to develop prediction models, it is much more limited in allowing unbiased assessment of provider diagnosis and management.[26] Providers may fail to document a UTI diagnosis in the EHR and antibiotics are often given for other diagnoses in patients with UTI symptoms. We therefore chose to compare the best-performing full and reduced models to 1) provider documentation of UTI diagnosis and 2) if the provider gave antibiotics OR documented a diagnosis of UTI, the provider was given credit for a UTI diagnosis. Cases where antibiotics were given and there was a clear alternative diagnosis (pneumonia, diverticulitis, colitis, cholecystitis, enteritis, obstruction, peritonitis, and cellulitis–captured by key word search) were not labeled as a UTI diagnosis. We believed examining provider UTI diagnosis alone would provide a reasonable upper bound for provider diagnostic specificity, and, likewise, a combination of UTI diagnosis or antibiotics for provider diagnostic sensitivity. Comparisons were performed for overall, admitted, and discharge cohorts. For these scenarios, we identified all medications prescribed or given within the ED meeting the ATC “infective” or “antibiotic” categories and urinary tract infection diagnoses by ICD9 and ICD10 codes (S2 Table).

### Model development

We developed models for UTI prediction using seven machine learning algorithms: random forest, extreme gradient boosting, adaptive boosting, support vector machine, elastic net, neural network, and logistic regression (R packages included: randomForest, xgboost, adaboost, e1071, glmnet, lme4, nnet, and caret). The first six algorithms were chosen for their ability to model nonlinear associations, resiliency to overfitting, relative ease in implementation, and general acceptance in the machine learning community. Logistic regression, commonly used in the medical field, was chosen as a baseline comparison. Data preprocessing steps, specified above, were common to all models. Models were developed using the full variable set (211 variables) and a reduced set of 10 variables selected through expert knowledge and literature review (Table 2). Expert and literature review-based selection was chosen over automated variable selection techniques to address user acceptance of model variables. Ten was chosen as a number that was felt to represent a reasonable upper threshold for development of an online calculator/app addressing usability concerns around manual data entry. Supported by prior literature, interaction terms were only assessed for selected urinalysis variables.[7, 9, 10] Where applicable, models were tuned through 10-fold cross validation and grid searches on respective hyperparameters within the training data set. All models were trained and validated on a randomly partitioned 80%/20% split of the data.

**Table 2:** Selected variables for reduced models.

Variable	References
Age	[3, 21]
Gender	[3, 6, 21]
UA Leukocytes	[3, 6, 10, 12, 21]
UA Nitrites	[3, 6, 10, 12, 21]
UA WBC	[3, 6, 10, 12, 21]
UA Bacteria	[3, 6, 10, 12, 21]
UA Blood	[3, 6, 10, 12, 21]
UA Epithelial Cells	[3, 6, 10, 12, 21]
History of UTI	[3, 6, 21]
Dysuria	[3, 6, 21]

#### Model comparison/Analysis

Descriptive statistics were used for baseline characteristics and outcomes. Univariate chi-square tests were used to compare categorical variables, and t-tests and ANOVA were used to compare continuous variables. We report the area under the curve (AUC) of the receiver operating characteristic (ROC) as the primary measure of model prediction. [27] AUC comparison was performed to evaluate significance via chi-square statistics using the method developed by Delong et al.[28] In order to account for multiple comparisons, a Bonferroni adjusted p-value of 0.004 was considered statistically significant. Additional statistics for comparison included sensitivity, specificity, positive and negative likelihood ratios with 95% confidence intervals (CI) and are reported at the optimal threshold for AUC.

For comparison to the two scenarios of clinical judgement, confusion matrices (i.e. 2x2 contingency matrices) were constructed. Sensitivity, specificity, and accuracy with 95%CI were calculated. The sensitivity is defined as the proportion of positive results out of the number of samples which were actually positive and specificity as the proportion of negative results out of the number of samples which were actually negative. Diagnostic accuracy was defined as the proportion of all tests that give a correct result. Exact binomial confidence limits were calculated for test sensitivity and specificity.[29] Confidence intervals for positive and negative likelihood ratios were based on formulae provided by Simel et al.[30] To increase interpretability, when comparing the models to UTI diagnosis alone, we set the specificity of the best performing models to that of UTI diagnosis allowing assessment of the differences in sensitivity. Similarly, when comparing the best performing models to UTI diagnosis OR antibiotic administration we set the sensitivity of each model to that of UTI diagnosis OR antibiotic administration allowing assessment of the differences in specificity. Differences in sensitivity and specificity between the models and proxies for provider judgement were analyzed using the adjusted Wald method and displayed with 95%CI.[31]

### Results

**Fig 1:**
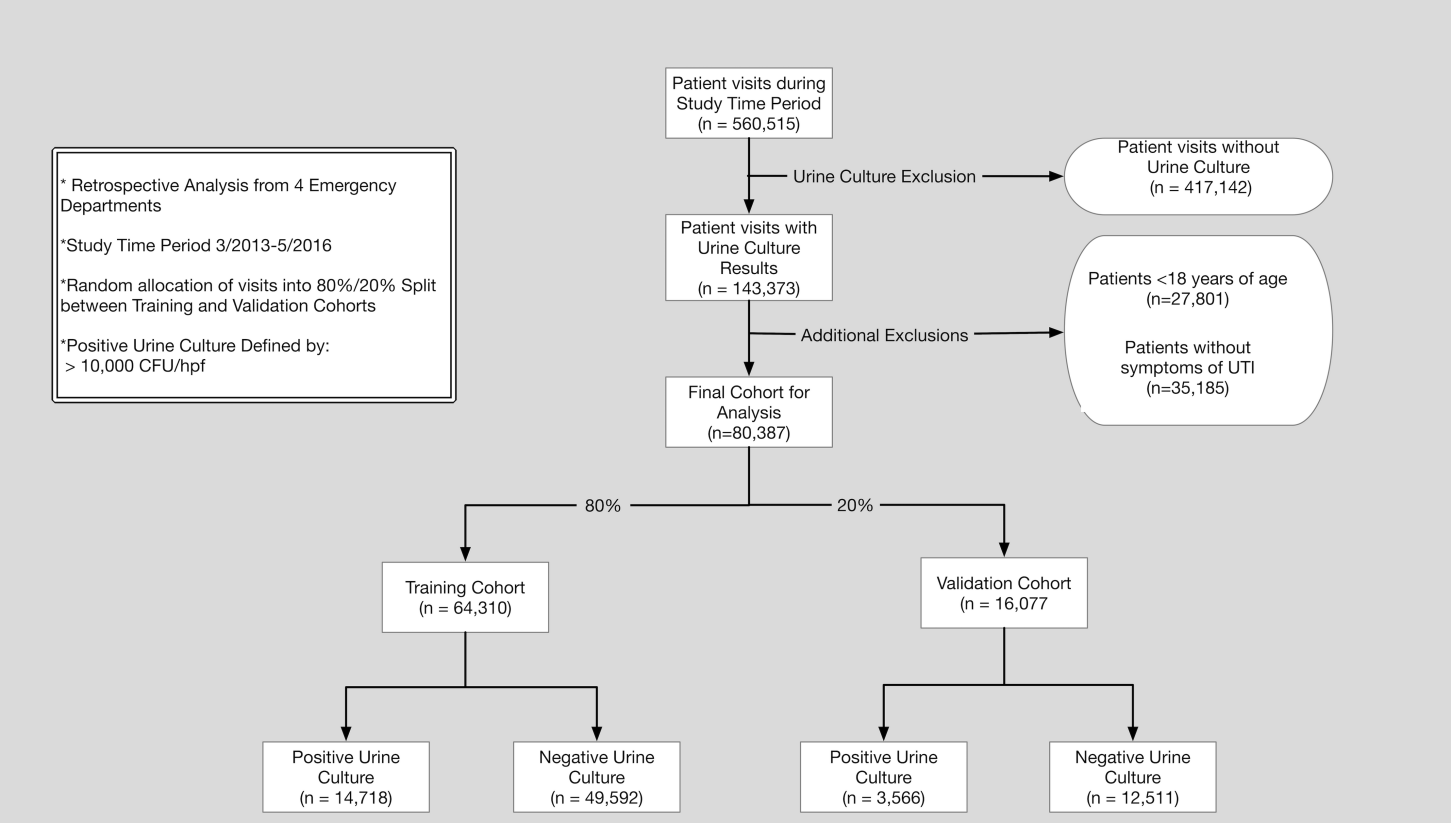
F*low diagram for study*.

During the study time period, there were 560,515 ED visits (410,173 patients). A total of 80,387 ED visits (55,365 patients) had urine culture results, symptoms potentially attributable to a UTI, and were ultimately included in the final analyses. There were 18,284 (23%) positive urine cultures, 14,335 (35%) in females, and 3,755 (18%) in males. Further demonstration of the training/validation cohorts and processing steps are demonstrated in Fig 1. The median age for the visits was 53 [IQR 34–72] and 68% were female. Additional basic demographic information and selected patient characteristics stratified by urine culture result are demonstrated in Table 3.

**Table 3:** 

	Urine Culture		
	Negative (n = 62,103)	Positive (n = 18284)	P-value
**Demographics**			
Age (median [IQR])	52.00 [33.00, 70.00]	58.00 [36.00, 79.00]	<0.001
Gender (%)—Female	40390 (65.0)	14335 (78.4)	<0.001
Race (%)			<0.001
White or Caucasian	33674 (54.2)	10202 (55.8)	
Black or African American	13093 (21.1)	3672 (20.1)	
Hispanic/Latino	1120 (1.8)	483 (2.6)	
Insurance status (%)			<0.001
Commercial	22057 (35.5)	5754 (31.5)	
Medicaid	18505 (29.8)	4907 (26.8)	
Medicare	16018 (25.8)	6381 (34.9)	
Self pay	671 (1.1)	128 (0.7)	
Other	3968 (6.4)	920 (5.0)	
Not Reported	884 (1.4)	194 (1.1)	
Arrival (%)			<0.001
Car	31834 (51.3)	9147 (50.0)	
EMS	19103 (30.8)	6744 (36.9)	
Walk-in	9026 (14.5)	1841 (10.1)	
Disposition (%)			<0.001
Admit	27588 (44.4)	8927 (48.9)	
Discharge	33579 (54.1)	9165 (50.2)	
**Past Medical History**			
Treated with Antibiotics	31411 (50.6)	14520 (79.4)	<0.001
Documented UTI Diagnosis	4152 (6.7)	6717 (36.7)	<0.001
Calculus of Urinary Tract	3887 (6.3)	1296 (7.1)	<0.001
Cancer	5263 (8.5)	1979 (10.8)	<0.001
Chronic Renal Failure	3082 (5.0)	1210 (6.6)	<0.001
Delirium and Cognitive Disorders	1970 (3.2)	1059 (5.8)	<0.001
Diabetes Mellitus	11261 (18.1)	4111 (22.5)	<0.001
Genitourinary Conditions	2924 (4.7)	1643 (9.0)	<0.001
HIV/AIDS	776 (1.2)	200 (1.1)	0.099
Hyperplasia of Prostate	1747 (2.8)	695 (3.8)	<0.001
Genital Disorders	1585 (2.6)	522 (2.9)	0.029
Paralysis	358 (0.6)	346 (1.9)	<0.001
Prolapse of Female Genital Organs	211 (0.3)	122 (0.7)	<0.001
Sexually Transmitted Infections	1010 (1.6)	281 (1.5)	0.417
Substance Related Disorders	2062 (3.3)	435 (2.4)	<0.001
History of Urinary Tract Infections	2764 (4.5)	2025 (11.1)	<0.001
Antineoplastics	2388 (3.8)	844 (4.6)	<0.001
Other immunosuppresants	1281 (2.1)	328 (1.8)	0.024
**Signs and Symptoms**			
Costoverterbral angle tenderness	2641 (4.3)	902 (4.9)	<0.001
Abdominal tenderness	25041 (40.3)	6060 (33.1)	<0.001
Back Pain	7481 (12.0)	1969 (10.8)	<0.001
Fatigue	10177 (16.4)	2865 (15.7)	<0.001
Fever	9923 (16.0)	3322 (18.2)	<0.001
Vaginal Bleeding	2368 (3.8)	598 (3.3)	<0.001
Vaginal Discharge	1353 (2.2)	360 (2.0)	<0.001
Abdoinal Pain	30896 (49.7)	6903 (37.8)	<0.001
Pelvic Pain	2292 (3.7)	551 (3.0)	<0.001
Flank Pain	6722 (10.8)	1913 (10.5)	0.226
Difficulty Urinating	1981 (3.2)	659 (3.6)	<0.001
Dysuria	6754 (10.9)	3553 (19.4)	<0.001
Hematuria	2873 (4.6)	1156 (6.3)	<0.001

**Fig 2:**
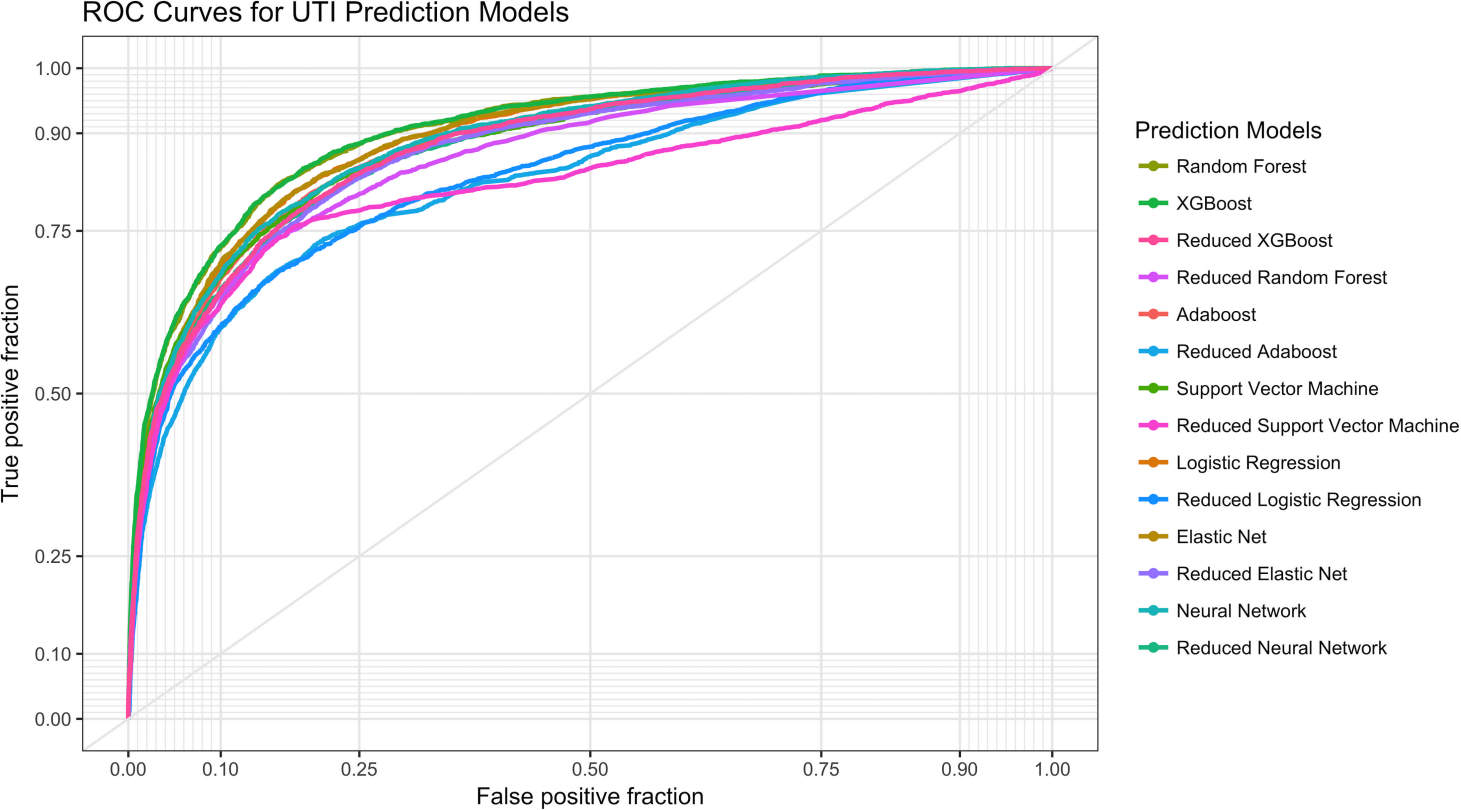
Receiver operating characteristic (ROC) curves for different machine learning models.

Classification results for the machine learning models are presented in Fig 2 and Table 4. The top classifier for the full models was XGBoost with an AUC of .904 (95%CI .898-.910) and was statistically better than all other models except Random Forest. The top classifier for the reduced models was XGBoost (AUC .877, 95%CI .871-.884). All full models were statistically better than the reduced models except for the reduced XGBoost model.

**Table 4:** Test characteristics of UTI prediction models on validation data*.

Models	AUC (95%CI)	Sensitivity (95% CI)	Specificity (95% CI)	+LR (95% CI)	-LR (95% CI)	Accuracy (95% CI)	P–value
**XGBoost**	**.904(.898-.910)**	61.7(60.0–63.3)	94.9 (94.5–95.3)	12.0(11.1–13.0)	.404(.387-.421)	87.5 (87.0–88.0)	NA
Random Forest	.902(.896-.908)	57.3(55.6–58.9)	96.0 (95.6–96.3)	14.3(13.0–15.6)	.445(.428-.462)	87.4 (86.9–87.9)	0.58
Adaboost	.880(.874-.887)	62.2(60.6–63.8)	92.3(91.8–92.7)	8.06(7.54–8.61)	.409(.392-.427)	85.6(85.1–86.2)	< .001
Support Vector Machine	.878(.871-.884)	49.6(47.9–51.2)	96.8(96.4–97.1)	15.3(13.8–16.9)	.521(.504-.538)	86.3(85.7–86.8)	< .001
ElasticNet	.892(.885-.898)	56.8(55.2–58.4)	94.9(94.5–95.2)	11.1(10.2–12.0)	.455(.438-.473)	86.4(85.9–87.0)	< .001
Logistic Regression	.891 (.884-.897)	57.5(55.8–59.1)	94.7(94.3–95.1)	10.9(10.0–11.8)	.449(.432-.466)	86.4(85.9–87.0)	< .001
Neural Network	.884 (.878-.890)	54.6(52.9–56.2)	95.3(95.0–95.7)	11.7(10.8–12.8)	.476(.460-.494)	86.3(85.8–86.8)	<001
**Reduced XGBoost**	**.877(.871-.884)**	54.7(53.0–56.3)	94.7(94.3–95.1)	10.4(9.6–11.3)	.479(.462-.496)	85.9(85.3–86.4)	< .001
Reduced Random Forest	.861(.853-.868)	54.8(53.1–56.4)	94.3(93.9–94.7)	9.66(8.94–10.4)	.479(.462-.497)	85.5(85.0–86.1)	< .001
Reduced Adaboost	.826(.817-.834)	61.9(60.3–63.5)	88.8(88.2–89.3)	5.50(5.21–5.81)	.429(.412-.448)	82.8(82.2–83.3)	< .001
Reduced Support Vector Machine	.822(.813-.832)	49.4(47.8–51.1)	95.8(95.4–96.1)	11.7(10.7–12.9)	.528(.511-.546)	85.5(84.9–86.0)	< .001
Reduced Elastic Net	.870(.863-.877)	52.4(50.7–54.1)	95.2(94.8–95.5)	10.9(9.99–11.8)	.500(.482-.571)	85.7(85.1–86.2)	< .001
ReducedLogistic Regression	.870(.863-.877)	53.3(51.6–54.9)	94.8(94.4–95.2)	10.3(9.52–11.2)	.492(.476-.510)	85.6(85.0–86.2)	< .001
Reduced Neural Network	.873(.867-.881)	54.0(52.3–55.6)	95.0(94.6–95.4)	10.9(10.0–11.8)	.485(.468-.502)	85.9(85.4–86.5)	< .001

* Test Characteristics determined at optimal AUC threshold

Full models were developed on 212 variables, while the reduced models were developed on 10 variables.

P-values obtained by AUC comparison to best performing model

In the validation cohort, 1616 (22.1%) admitted visits and 1712 (20.1%) discharge visits were diagnosed with UTI. Within this cohort, the number of admit and discharge visits with a documented diagnosis of UTI receiving antibiotics was 1610 (99.6%) and 1693 (98.9%), respectively. Comparison of the top-performing (XGBoost) model with provider diagnosis and antibiotic prescribing are presented in the form of confusion matrices with associated sensitivities, specificities, accuracies, and differences (Tables 5 and 6). While setting the sensitivity of the best-performing models to the same value as the combination of antibiotics OR documentation of UTI diagnosis, the best performing full and reduced model demonstrated far superior specificity with a 33.3 (31.3–34.3) and 29.6 (28.5–30.6) difference, respectively. Framed within a more clinical perspective, in applying the model to the overall validation admitted/discharge cohort approximately 1 in 4 patients (4109/15855) would be re-categorized from a false positive to a true negative when compared to provider judgement as determined by UTI diagnosis and antibiotic prescribing. Comparing only UTI diagnosis to the best performing models set at the same specificity, the best performing full and reduced model also demonstrated far superior sensitivity with a 38.7 (38.1–39.4) and 33.2 (32.5–33.9) difference, respectively. In the overall validation admitted/discharge cohort approximately 1 in 11 patients (1372/15855) would be re-categorized from a false negative to a true positive when compared to provider judgement as determined by UTI diagnosis alone. Among admit visits receiving antibiotics, there were 156 visits (13.2%) with clear alternative infectious diagnoses in those with positive urine cultures and 529 (21.3%) in those with negative urine cultures. Among discharge visits who received antibiotics, there were 52 (4.3%) visits with clear alternative infectious diagnoses and 200 (9.0%) in those with negative urine cultures.

**Table 5:** Comparison of provider judgment (UTI diagnosis or antibiotic administration) to best performing models for prediction of urine culture results.

	Model	TP	FN	TN	FP	Sens (95%CI)	Spec (95%CI	Acc (95%CI)	Diff Spec (95%)
**Overall (Admit and** **Discharge)**									
	Antibiotics or UTI diagnosis	2601	923	6879	5434	73.8 (72.3–75.2)	55.9 (55.1–56.8)	59.9 (59.1–60.6)	NA
	XGBoost	2601	923	10988	1325	73.8 (72.3–75.2)	89.2(88.6–89.8)	85.8(85.3–86.3)	33.3 (31.3–34.3)
	Reduced XGBoost	2601	923	10529	1784	73.8 (72.3–75.2)	85.5(84.9–86.1)	82.9(82.3–83.5)	29.6 (28.5–30.6)
**Admitted**									
	Antibiotics or UTI diagnosis	1344	396	2567	3004	77.7 (75.1–79.2)	46.1 (44.8–47.4)	53.5 (52.3–54.6)	NA
	XGBoost	1344	396	5055	516	77.7 (75.1–79.2)	90.7(89.9–91.5)	87.5 (86.7–88.3)	44.6 (43.4–45.8)
	Reduced XGBoost	1344	396	4820	751	77.7 (75.1–79.2)	86.5 (85.6–87.4)	84.3(83.5–85.1)	40.4 (39.3–41.6)
**Discharged**									
	Antibiotics or UTI diagnosis	1257	527	4312	2430	70.4 (68.3–72.6)	64.0 (62.8–65.1)	65.3 (64.2–66.3)	NA
	XGBoost	1257	527	5933	809	70.4 (68.3–72.6)	88.0 (87.2–88.8)	84.3 (83.5–85.1)	24.0(22.8–25.1)
	Reduced XGBoost	1257	527	5709	1033	70.4 (68.3–72.6)	84.7(83.8–85.5)	81.7(80.8–82.5)	20.7(19.5–21.9)

In order to demonstrate the additive value of the models, each predictive model threshold was set to same sensitivity as provider judgment (UTI diagnosis or Antibiotic Administration) and examined for its ability to predict urine culture results.

TP = True Positive, FN = False Negative, TN = True Negative, FP = false positive, Sens = Sensitivity, Spec = Specificity, Acc = Accuracy

Diff spec = difference in specificity between the model and provider judgment 95%CI

**Table 6:** Comparison of provider judgment (UTI diagnosis) to best performing models for prediction of urine culture results.

	Model	TP	FN	TN	FP	Sens (95%CI)	Spec (95%CI	Acc (95%CI)	Diff Sens (95%)
**Overall**									
	UTI diagnosis	1447	2077	10432	1881	41.3 (39.7–42.9)	84.7 (84.1–85.4)	75.1 (74.4–75.8)	NA
	XGBoost	2819	705	10432	1881	80.0 (78.6–81.3)	84.7 (84.1–85.4)	83.7 (83.1–84.2)	38.7 (38.1–39.4)
	Reduced XGBoost	2626	898	10432	1881	74.5 (73.0–75.9)	84.7 (84.1–85.4)	82.5 (81.9–83.0)	33.2 (32.5–33.9)
**Admitted**									
	UTI diagnosis	652	1088	4607	964	37.4 (35.2–39.8)	82.7 (81.7–83.7)	71.9 (70.9–73.)	NA
	XGBoost	1502	238	4607	964	86.3 (84.6–87.9)	82.7 (81.7–83.7)	83.6 (82.7–84.4)	48.9 (47.7–49.1)
	Reduced XGBoost	1414	326	4607	964	81.3 (79.4–83.1)	82.7 (81.7–83.7)	82.4 (81.7–83.7)	43.9 (42.6–45.1)
**Discharged**									
	UTI diagnosis	795	989	5825	917	44.6 (42.2–46.9)	86.4 (85.5–87.2)	77.6 (76.7–78.5)	NA
	XGBoost	1317	467	5825	917	73.8 (71.7–75.9)	86.4 (85.5–87.2)	83.8 (83.0–84.5)	29.2 (28.0–30.4)
	Reduced XGBoost	1212	572	5825	917	67.9 (65.7–70.1)	86.4 (85.5–87.2)	82.5 (81.7–83.3)	23.3 (22.1–24.5)

In order to demonstrate the additive value of the models, each predictive model threshold was set to the same specificity as provider judgment (UTI diagnosis) and examined for its ability to predict urine culture results.

TP = True Positive, FN = False Negative, TN = True Negative, FP = false positive, Sens = Sensitivity, Spec = Specificity, Acc = Accuracy, Diff Sens = difference in specificity between the model and provider judgment 95%CI

## Discussion

In this retrospective observational study of urinary tract infections, a common ED diagnosis with high rates of diagnostic error, we used machine learning algorithms and a large dataset to accurately diagnose positive urine culture results. The top-performing algorithm, XGBoost, achieved an AUC of .904(.898-.910), and overall accuracy of 87.5% (95%CI 87.0–88.0), almost ten percentage points higher accuracy than the best performing model in the literature.[16] Even for models trained on a more limited set of variables, the best models achieved excellent results with an AUC of .877(.871-.884) and an accuracy of 85.9%(95%CI 85.3–86.4). In comparison to proxies of provider judgment, the best performing models were far more specific than a combination of antibiotics OR documentation of UTI diagnosis and far more sensitive than documentation of UTI diagnosis alone.

Previous studies developing predictive models for UTI are limited by small data sets, poor generalizability to the ED, and diagnostic performance. [12–17] The idea that a predictive model would be useful for UTI diagnosis in the ED has been around for some time. Wigton et al. in 1985 developed a scoring model (derived from discriminant analysis) based on history, physical, and laboratory in 248 female patients in the ED with validation on 298 patients.[32] In this study the prevalence of UTI was 61% and the reported AUC was 0.78, accuracy 74%, sensitivity 93%, and specificity 44%. This is the only model developed on ED patients of which we are aware. Subsequent models, almost all some form of clinical decision rule on a few variables, were developed predominantly in outpatient settings on several hundred patients with prevalence values of 53–62% and generally did not have separate validation data sets.[7] Accuracy for these studies was 67–76% with sensitivity values of 64.9–82.0% and specificity values of 53.7–94.8%. The best performing model we found in the literature was by Heckeling et al. and used neural networks with a genetic algorithm for variable selection.[16] The model by Heckerling et al. was developed in an outpatient setting on 212 female patients and had an AUC of 0.78, and accuracy of 78%, but lacked testing on a separate validation data set. Our models, in contrast, were developed on a data set approximately 100 times in size, utilizing hundreds of variables and machine learning algorithms on a diverse set of ED patients. We achieved a top-performing AUC 0.12 points higher than Wigton et al. and Heckerling et al. with 9–12% greater accuracy. The reduced models, while generally not performing as well as the full models, still achieved much higher results than previously reported models and decision aids.

A model that fails to indicate an ability to improve current care has little value, regardless of its predictive ability, and recent evidence suggests that most clinical decisions rules fail to outperform clinical judgement.[33] In examining the literature, only one of the prior models for UTI prediction demonstrated its potential clinical impact.[14] McIsaac et al. showed that with implementation of their simple decision aid unnecessary antibiotics would be reduced by 40.2%. Recognizing the limitations of EHR data and retrospective analysis, we chose to compare the models to two proxies for provider judgment, 1) the provider was considered to have diagnosed the patient with a UTI if, and only if, the diagnosis was documented—optimizing specificity, and 2) if the provider gave antibiotics or diagnosed the patient with UTI the provider was given credit for a UTI diagnosis, thus optimizing sensitivity. These scenarios are “optimal” from the provider standpoint in that it is likely that a portion of visits which eventually have a positive urine culture patients were given antibiotics for some other suspected cause and that in visits with an eventual negative urine culture there is a portion of patients who did not have a documented UTI, but the provider nevertheless likely had that diagnosis in mind (e.g. patient diagnosed with dysuria and given antibiotics but eventual urine culture is negative). In comparison to these proxies of provider judgment, the best performing models were far more specific than a combination of antibiotics OR documentation of UTI diagnosis and far more sensitive than documentation of UTI diagnosis alone. This was true in both discharge and admit visits with the larger difference in admit visits possibly a consequence of a lower threshold for antibiotic administration, complexity of presentation, and higher acuity visits. Moreover, even in a theoretical scenario where provider judgement is assigned both optimal bounds (sensitivity assigned from UTI or antibiotics scenario– 73.8% and specificity assigned from the UTI diagnosis only scenario– 84.7%), both the full and reduced models still demonstrate overall superior performance. Viewed from another perspective, our findings suggest that implementation of the algorithm has the potential to greatly reduce the number of false positives and false negatives for UTI diagnosis. For example, in the overall cohort (both discharged and admitted patients) approximately 1 in 4 patients (4111/15855) were re-categorized from a false positive to a true negative when comparing XGBoost to antibiotics OR documentation of UTI diagnosis.

Advances in machine learning, coupled with training on large EHR datasets, have the ability to disrupt the areas of diagnosis and prognosis in emergency medicine.[34] Already in other fields, expert level, or above expert level, performance has been achieved in areas as diverse as the diagnosis of diabetic retinopathy[35] and heart failure prediction.[36] UTI diagnosis is an area particular ripe for improvement through machine learning based clinical decision support. UTI diagnosis has a high error rate, the primary information that is used for diagnosis are abstract lab values with multiple categories, and there is a lack of reinforcement learning (ED providers rarely see the final culture results). Incorporation of machine learning algorithms into existing workflows, however, is not without difficulty. Models that use hundreds of variables make manual entry unfeasible and are currently difficult to “hard” code within EHR platforms/databases or to export to 3^rd^ party applications. Progress is being made in this area with tools incorporating the predictive modeling markup language (PMML) facilitating interoperable exchange of models.[37] Importantly, for UTI diagnosis, our results suggest using a reduced model in, for example, an online app would result in only a small performance loss compared to the full model and still significantly improve diagnostic accuracy. The app could incorporate pretest probabilities of disease facilitating personalized decisions for each patient based on patient/doctor determined testing and treatment thresholds. Future implementation studies could then examine the effect of clinical decision support system app on diagnostic error and outcomes.

### Limitations

The current study has several limitations. First, we recognize that without prospectively collecting data on clinical diagnosis, uncertainty exists regarding the performance of clinical judgement in our study. We, however, believe that the scenarios examined serve to minimize this risk. Second, there is currently no clear accepted level for a positive urine culture with a range in the literature from 10^2 cfu/mL to 10^5 cfu/mL. [12–17] Conceivably different thresholds would result in different test performances. Our choice of 10^4 cfu/mL is a middle ground and was unable to be adjusted due to standardized laboratory reporting within the EHR. Third, our model was built on data from a single healthcare institution within a confined geographic region and would require further validation at other institutions prior to implementation at those sites. Alternately, institutions could take the methods and variables used here and build their own models. Fourth, our data only included visits with urine culture results limiting its extension to patients who may have only had urinalysis or urine dipstick test. Last, our approach was limited to data elements available during each ED visit and does not include unstructured data elements, such as features in clinical notes, that may further improve the predictive accuracy.

### Conclusion

In this study developing and validating models for prediction of urinary tract infections in emergency department visits on a large EHR dataset, the best performing machine learning algorithm, XGBoost, accurately diagnosed positive urine culture results, and outperformed previously developed models in the literature and several proxies for provider judgment. Futures implementation studies should prospectively examine the impact of the model on outcomes and diagnostic error.

## Supporting information

S1 File Minimal data set. Minimal Data set necessary for analyses.
(CSV)

S2 File Code for analysis. R code for analyses. Please see code for further description.
(R) Click here for additional data file.

S1 Table Variable list. Full variable list for machine learning models.
(DOCX)

S2 Table UTI diagnoses. ICD codes for UTI.
(DOCX)
