# Supplementary material for: Predicting urinary tract infections in the emergency department with machine learning
Source: PLoS One. 2018 Mar 7;13(3):e0194085. doi: 10.1371/journal.pone.0194085 (PMC5841824; doi:10.1371/journal.pone.0194085)
Supplement: S1 Table — Full variable list for machine learning models. (DOCX) [file pone.0194085.s003.docx]

| Category | Variable |
| --- | --- |
| Urinalysis |  |
|  | "ua_bacteria" |
|  | "ua_bili" |
|  | "ua_blood" |
|  | "ua_clarity" |
|  | "ua_color" |
|  | "ua_epi" |
|  | "ua_glucose" |
|  | "ua_ketones" |
|  | "ua_leuk" |
|  | "ua_nitrite" |
|  | "ua_ph" |
|  | "ua_protein" |
|  | "ua_rbc" |
|  | "ua_spec_grav" |
|  | "ua_urobili" |
|  | "ua_wbc" |
| Physical Findings |  |
|  | "CVA_tenderness" |
|  | "abd_tenderness" |
|  | "abd_soft" |
|  | "abd_distended" |
|  | "abd_gaurding" |
|  | "abd_mass" |
|  | "abd_rebound" |
|  | "abd_rigidity" |
|  | "back_pain" |
|  | "fatigue" |
|  | "fever" |
|  | "vag_bleeding" |
|  | "vag_discharge" |
|  | "abd_distended2" |
|  | "abd_pain" |
|  | "gen_neg" |
|  | "pelvic_pain" |
|  | "alert" |
|  | "ams" |
|  | "weakness" |
|  | "oriented" |
|  | "psychiatric_confusion" |
|  | "flank_pain" |
|  | "dec_urine_vol" |
|  | "diff_urinating" |
|  | "dysuria" |
|  | "hematuria" |
|  | "polyuria" |
| Demographics/Arrival Info |  |
|  | "chief_complaint" |
|  | "age" |
|  | "gender" |
|  | "race" |
|  | "ethnicity" |
|  | "lang" |
|  | "maritalStatus" |
|  | "employStatus" |
|  | "insurance_status" |
|  | "arrival" |
| Vitals |  |
|  | "Temp_First" |
|  | "Temp_Last" |
|  | "Temp_Max" |
|  | "Temp_Min" |
|  | "Temp_Mean" |
|  | "HR_First" |
|  | "HR_Last" |
|  | "HR_Max" |
|  | "HR_Min" |
|  | "HR_Mean" |
|  | "SBP_First" |
|  | "SBP_Last" |
|  | "SBP_Max" |
|  | "SBP_Min" |
|  | "SBP_Mean" |
|  | "DBP_First" |
|  | "DBP_Last" |
|  | "DBP_Max" |
|  | "DBP_Min" |
|  | "DBP_Mean" |
|  | "RR_First" |
|  | "RR_Last" |
|  | "RR_Max" |
|  | "RR_Min" |
|  | "RR_Mean" |
|  | "O2_Sat_First" |
|  | "O2_Sat_Last" |
|  | "O2_Sat_Max" |
|  | "O2_Sat_Min" |
|  | "O2_Sat_Mean" |
|  | "O2_Amount_First" |
|  | "O2_Amount_Last" |
|  | "O2_Amount_Max" |
|  | "O2_Amount_Min" |
|  | "O2_Amount_Mean" |
|  | "O2_Dependency_First" |
|  | "O2_Dependency_Last" |
|  | "GCS_First" |
|  | "GCS_Last" |
| Labs |  |
|  | "Absolute_Lymphocyte_Count" |
|  | "Alanine_Aminotransferase" |
|  | "Alkaline_Phosphatase" |
|  | "ANC" |
|  | "Anion_Gap" |
|  | "Aspartate_Aminotransferase" |
|  | "Basophils" |
|  | "BUN" |
|  | "Calcium" |
|  | "Chloride" |
|  | "CO2" |
|  | "Creatinine" |
|  | "Eosinophils" |
|  | "Glucose" |
|  | "Hematocrit" |
|  | "Hemoglobin" |
|  | "Lymphocytes" |
|  | "MCH" |
|  | "MCHC" |
|  | "MCV" |
|  | "Monocytes" |
|  | "MPV" |
|  | "Neutrophils" |
|  | "Platelets" |
|  | "Potassium" |
|  | "RBC" |
|  | "RDW" |
|  | "Sodium" |
|  | "WBC" |
| Past Medical/Surgical History |  |
|  | "Abdominal_hernia" |
|  | "Abdominal_pain" |
|  | "Acute_and_unspecified_renal_failure" |
|  | "Anal_and_rectal_conditions" |
|  | "Calculus_of_urinary_tract" |
|  | "Cancer" |
|  | "Chronic_renal_failure" |
|  | "Congestive_heart_failure__nonhypertensive" |
|  | "Coronary_atherosclerosis_and_other_heart_disease" |
|  | "Delirium__dementia__and_amnestic_and_other_cognitive_disorders" |
|  | "Diabetes_mellitus_with_complications" |
|  | "Diabetes_mellitus_without_complication" |
|  | "Diabetes_or_abnormal_glucose_tolerance_complicating_pregnancy__childbirth__or_the_puerperium" |
|  | "Genitourinary_congenital_anomalies" |
|  | "Genitourinary_symptoms_and_ill_defined_conditions" |
|  | "Heart_valve_disorders" |
|  | "HIV_infection" |
|  | "Hodgkins_disease" |
|  | "Hyperplasia_of_prostate" |
|  | "Inflammatory_conditions_of_male_genital_organs" |
|  | "Leukemias" |
|  | "Maintenance_chemotherapy__radiotherapy" |
|  | "Menstrual_disorders" |
|  | "Nephritis__nephrosis__renal_sclerosis" |
|  | "Non_Hodgkins_lymphoma" |
|  | "Other_and_ill_defined_heart_disease" |
|  | "Other_female_genital_disorders" |
|  | "Other_male_genital_disorders" |
|  | "Other_non_epithelial_cancer_of_skin" |
|  | "Pancreatic_disorders__not_diabetes_" |
|  | "Paralysis" |
|  | "Parkinsons_disease" |
|  | "Poisoning_by_nonmedicinal_substances" |
|  | "Prolapse_of_female_genital_organs" |
|  | "Pulmonary_heart_disease" |
|  | "Screening_and_history_of_mental_health_and_substance_abuse_codes" |
|  | "Septicemia__except_in_labor_" |
|  | "Sexually_transmitted_infections__not_HIV_or_hepatitis_" |
|  | "Substance_related_disorders" |
|  | "Urinary_tract_infections" |
| Outpatient Medications |  |
|  | "ANALGESIC_AND_ANTIHISTAMINE_COMBINATION" |
|  | "ANALGESICS" |
|  | "ANESTHETICS" |
|  | "ANTI_OBESITY_DRUGS" |
|  | "ANTIALLERGY" |
|  | "ANTIARTHRITICS" |
|  | "ANTIASTHMATICS" |
|  | "ANTIBIOTICS" |
|  | "ANTICOAGULANTS" |
|  | "ANTIDOTES" |
|  | "ANTIFUNGALS" |
|  | "ANTIHISTAMINE_AND_DECONGESTANT_COMBINATION" |
|  | "ANTIHISTAMINES" |
|  | "ANTIHYPERGLYCEMICS" |
|  | "ANTIINFECTIVES" |
|  | "ANTIINFECTIVES_MISCELLANEOUS" |
|  | "ANTINEOPLASTICS" |
|  | "ANTIPARKINSON_DRUGS" |
|  | "ANTIPLATELET_DRUGS" |
|  | "ANTIVIRALS" |
|  | "AUTONOMIC_DRUGS" |
|  | "BIOLOGICALS" |
|  | "BLOOD" |
|  | "CARDIAC_DRUGS" |
|  | "CARDIOVASCULAR" |
|  | "CNS_DRUGS" |
|  | "COLONY_STIMULATING_FACTORS" |
|  | "CONTRACEPTIVES" |
|  | "COUGH_COLD_PREPARATIONS" |
|  | "DIAGNOSTIC" |
|  | "DIURETICS" |
|  | "EENT_PREPS" |
|  | "ELECT_CALORIC_H2O" |
|  | "GASTROINTESTINAL" |
|  | "HERBALS" |
|  | "HORMONES" |
|  | "IMMUNOSUPPRESANT" |
|  | "INVESTIGATIONAL" |
|  | "MISCELLANEOUS_MEDICAL_SUPPLIES__DEVICES__NON_DRUG" |
|  | "MUSCLE_RELAXANTS" |
|  | "PRE_NATAL_VITAMINS" |
|  | "PSYCHOTHERAPEUTIC_DRUGS" |
|  | "SEDATIVE_HYPNOTICS" |
|  | "SKIN_PREPS" |
|  | "SMOKING_DETERRENTS" |
|  | "THYROID_PREPS" |
|  | "UNCLASSIFIED_DRUG_PRODUCTS" |
|  | "VITAMINS" |
