# Supplementary material for: Predicting urinary tract infections in the emergency department with machine learning
Source: PLoS One. 2018 Mar 7;13(3):e0194085. doi: 10.1371/journal.pone.0194085 (PMC5841824; doi:10.1371/journal.pone.0194085)
Supplement: S2 Table — ICD codes for UTI. (DOCX) [file pone.0194085.s004.docx]

List of ICD9, ICD10 codes for Urinary Tract Infection

595.0 Acute cystitis ICD9CM

595.9 Cystitis, unspecified ICD9CM

599.0 Urinary tract infection, site not specified ICD9CM

N30.00 Acute cystitis without hematuria ICD10CM

N30.01 Acute cystitis with hematuria ICD10CM

N30.80 Other cystitis without hematuria ICD10CM

N30.81 Other cystitis with hematuria ICD10CM

N30.90 Cystitis, unspecified without hematuria ICD10CM

N30.91 Cystitis, unspecified with hematuria ICD10CM

N39.0 Urinary tract infection, site not specified ICD10CM

590.80 Pyelonephritis ICD9

O23.01 Pyelonephritis O23.01 ICD10

N10, N12, N13 Pyelonephritis ICD10
